# Solidified Salt Melts of the NaCl–KCl–CeF_3_–EuF_3_ System as Promising Luminescent Materials

**DOI:** 10.3390/ma17225565

**Published:** 2024-11-14

**Authors:** Viktor Zinchenko, Ganna Volchak, Nataliia Chivireva, Pavlo Doga, Yaroslav Bobitski, Oleh Ieriomin, Serhii Smola, Anton Babenko, Małgorzata Sznajder

**Affiliations:** 1 O. Bogatsky Physico-Chemical Institute NASU, Lustdorfska Doroga 86, 65080 Odesa, Ukraine; vfzinchenko@ukr.net (V.Z.); dogapavel@gmail.com (P.D.); sssmola@gmail.com (S.S.); anton.octane.sr@gmail.com (A.B.); 2 Institute of Physics, University of Rzeszow, Pigonia 1, 35-310 Rzeszow, Poland; ybobytskyy@ur.edu.pl (Y.B.); msznajder@ur.edu.pl (M.S.)

**Keywords:** cerium fluoride, europium fluoride, NaCl–KCl melt, solidified solution-melt, oxidation–reduction, photoluminescence

## Abstract

This study presents the results of investigating the interaction between the CeF₃–EuF₃ system and the NaCl–KCl salt melt using spectroscopic methods. It was found that CeF₃ ions undergo no significant changes upon dissolution in the NaCl–KCl melt. In contrast, the dissolution of EuF₃, both individually and within the CeF₃–EuF₃ system, is accompanied by redox reactions leading to the formation of Eu^2^⁺. The diffuse reflectance spectra of both the bottom (insoluble sediment) and upper parts of the solidified salt melt in the UV range indirectly indicate photoluminescence excitation from Ce^3^⁺ and Eu^2^⁺ ions. In addition, absorption bands in the near-IR region (1900–2300 cm⁻^1^) confirm the retention of some Eu^3^⁺ ions in the salt melt. The study explored the effects of various factors—including sample composition, excitation wavelength, prior and subsequent heat treatment, and medium composition—on the excitation and emission spectra of the samples. Intense 5d-4f luminescence of Ce^3^⁺ and Eu^2^⁺ ions (at 330 and 430 nm, respectively) was observed predominantly in the upper part of the salt melts, along with much weaker 4f-4f luminescence from Eu^3^⁺ ions. Certain parameters were optimized to reduce the luminescence contribution from Ce^3^⁺ and especially Eu^3^⁺ ions while enhancing the luminescence of Eu^2^⁺ ions. Solidified salt solution-melts of the NaCl–KCl–CeF₃–EuF₃ system show promise as materials for developing solar ultraviolet radiation detectors.

## 1. Introduction

The sun is a source of various types of radiation, including visible light, thermal (infrared) radiation, ultraviolet radiation, and X-ray radiation. Due to the Earth’s atmosphere, a significant portion of high-energy radiation, particularly ultraviolet radiation, does not reach the surface. The radiation that penetrates the lower layers of the atmosphere is categorized into three bands: UVA (315–400 nm), UVB (280–315 nm), and UVC (200–280 nm). The ozone layer significantly attenuates the low-energy portion of the UVC and UVB bands, resulting in pronounced fluctuations in radiation intensity within the range of 250 to 410 nm.

Sunlight reaching the Earth’s surface exhibits a specific spectral intensity dependence, as illustrated in [1]. In the range of 315 to 375 nm, the intensity of the sun’s ultraviolet radiation is nearly independent of the wavelength. To measure the intensity (flux) of ultraviolet radiation, thermoluminescence methods are promising, as the integrated area corresponds to the concentration of accumulated charge in the dosimeter material [2]. The review [3] analyzes the latest developments in ultraviolet radiation detectors. Notable among these are photoconductive-type detectors, Schottky barrier-type detectors, (metal–semiconductor–metal)-type detectors, and several modern developments based on nanostructured materials.

Cerium and europium are lanthanides with variable valence, as they can exist in multiple oxidation states. In addition to the +3 oxidation state, which is commonly encountered in their compounds, particularly fluorides, they can also exhibit +4 and +2 oxidation states under specific conditions.

Generally, Eu^2^⁺ and Ce^3^⁺ ions are more stable in a reducing atmosphere, while the stability of Eu^3^⁺ and Ce⁴⁺ is favored in an oxidizing environment. The luminescent properties of Eu^2^⁺ and Ce^3^⁺ are primarily determined by 5d-4f electronic transitions, making them strongly dependent on the surrounding environment, including factors such as symmetry, coordination number, and crystal field strength. This dependence arises because the excited 5d state is not shielded by 5s^2^ and 5p⁶ electrons [4,5]. Conversely, Eu^3^⁺ ions exhibit orange-red luminescence due to 4f-4f electronic transitions, specifically from 5D₀ → 7F₂ at a wavelength of 617 nm, which has been widely utilized in industrial red phosphors such as Y_2_O_3_:Eu^3+^ and Y_2_O_2_S:Eu^3+^. The authors of [6,7] have established the possibility of coexisting different-valent forms of europium (II, III) and cerium (III, IV) within a single matrix, along with the potential for energy transfer between these different forms.

It was found in paper [8] that halides doped with europium exhibit thermoluminescence signals when excited by radiation with wavelengths ranging from 220 to 280 nm. This phenomenon has been utilized to detect solar UVC radiation from KCl:Eu^2+^ single crystals, which serve as thermoluminescent dosimeters. The effectiveness of the KCl:Eu^2+^ system as a selective UVC dosimeter is described in references [9,10]. The TL excitation spectrum of KCl:Eu^2+^ single crystals was measured, and its convolution with the solar spectral radiation reaching the Earth was estimated using a radiation transfer model. By comparing the thermoluminescence sensitivity of KCl:Eu^2+^ to the measured dose of solar radiation, the authors concluded that although UVC radiation at ground level is six orders of magnitude less than UVB radiation, it is, nevertheless, detectable and can be measured using KCl:Eu^2+^ dosimeters.

In turn, the authors of [11] simulated the flow of solar ultraviolet radiation registered by a dosimeter throughout the day. They confirm that the Eu^2^⁺-doped KCl crystal behaves under sunlight as a narrow-band Gaussian detector centered at a wavelength of 265 nm. Furthermore, a comparison between the KCl:Eu^2+^ system and commercially available broad-band UVB biological sensors (biometers) indicates that europium-doped crystals are more sensitive to small changes in solar UVB flux, making them a suitable choice for detecting significant ozone depletion.

The study of optical phenomena in KCl single crystals doped with Ce^3^⁺ was conducted in [12]. The observed blue glow is attributed to the emission of Ce^3^⁺ ions, indicating their involvement in the thermoluminescence process. The authors note the low concentration of Ce^3^⁺ ions in the KCl matrix, which reduces the efficiency of this process. Article [13] presents results on the optical characteristics of KCl single crystals co-doped with Eu^2^⁺ and Ce^3^⁺. It is noted that co-doped crystals exhibit increased thermoluminescence intensity compared to the samples doped with only one of the ions. These results confirm the phenomenon of energy transfer between Ce^3^⁺ and Eu^2^⁺ in the KCl matrix, highlighting the potential for using this material in the development of thermally stimulated dosimeters.

However, the necessity of using KCl single crystals, which are laboriously grown, as a matrix with the incorporation of highly hygroscopic compounds (EuCl₂ and CeCl₃) that are prone to hydrolysis and oxidation in air poses a significant barrier to the broader application of such materials. Consequently, some authors have shifted their focus to eutectic materials as potential matrices for luminescent dosimeters. For instance, the authors in [14] proposed the use of self-organized eutectics and systems based on them as scintillation materials for ionizing radiation detectors.

Interesting results regarding halide melts containing europium are presented in [15]. It was established that both ionic and electronic conductivity are observed in the NaCl–KCl melt containing Eu(II) and Eu(III) complexes. Additionally, the authors of [16] measured the electronic absorption spectra of Nd^3^⁺ incorporated into LiCl–KCl and NaCl–CsCl eutectics.

Considering the variety of UV radiation detectors and the promising materials for these detectors, it is crucial to systematically measure the UV radiation flux reaching the surface of our planet. This is especially important given the potential for a significant increase in the UV radiation flux. Therefore, there is a pressing need to develop durable and reliable sensors for monitoring incoming solar ultraviolet radiation. One promising method for synthesizing detection materials is the use of self-organizing eutectics and systems based on eutectics, particularly NaCl–KCl.

We previously investigated the solubility of lanthanide fluorides (La÷Lu) in an equimolar salt melt of NaCl–KCl and the potential for detecting them using diffuse reflectance spectroscopy and the luminescence of solidified melts [17]. For the first time, we observed the course of redox reactions between EuF_3_ and the components of the salt melt, leading to the transformation of Eu(III) into Eu(II). This transformation was evidenced by the almost complete disappearance of the absorption band characteristic of EuF_3_ in the range of 1900–2200 nm and the emergence of luminescence in the range characteristic of Eu^2+^ at 425–430 nm [18]. A similar phenomenon was observed in the solidified melt of NaCl–KCl with CeF_3_–EuF_3_ [19].

The primary goal of the present paper is to conduct a detailed study of the effect of CeF_3_ on the luminescence characteristics of Eu^2+^ and Eu^3+^ ions in the NaCl–KCl melt. Additionally, this work aims to optimize the ratio between CeF_3_ and EuF_3_, as well as the CeF_3_–EuF_3_ mixture within the NaCl–KCl solidified salt melt. Furthermore, we seek to optimize the heat treatment method to enhance the emission efficiency of Eu^2+^ ions in the salt melt.

## 2. Materials and Methods

### 2.1. Manufacturing of Cerium and Europium Fluorides and the NaCl–KCl Salt System

The synthesis of samples in the CeF₃–EuF₃ system, with molar ratios of 1:1, 2:1, and 1:2 between CeF₃ and EuF₃, and mass ratios of 1:9 (in the first case) and 2:8 (in the second case) between fluoride systems and the NaCl–KCl salt system, was conducted using the melt method. For this purpose, finely ground powders—obtained by thorough grinding in a mortar to achieve a minimum particle size of approximately 1 μm—were used. According to Kortüm et al. [20], particles of 1 μm or larger exhibit optical properties that are nearly independent of grain size, whereas size effects, such as a “blue shift”, only begin to appear in nanoparticles (<100 nm). The average crystallite size was determined from XRD data using the Scherrer equation, yielding a value of 131.8 nm.

(1) $$D=\frac{K\cdot \lambda }{\beta \cdot \cos\theta },$$

where the FWHM values of the peaks were used to estimate the crystallite size. According to the Debye–Scherrer equation, *D* ≤ 200 nm represents the average crystallite size, with the Scherer constant *K* ranging from 0.68 to 2.08 (typically 0.94 for spherical crystallites with cubic symmetry). Here, *λ* is the X-ray wavelength (CuKα = 1.5406 Å), *β* is line broadening at FWHM in radians, and *θ* is the Bragg angle in degrees (half of 2*θ*).

The powders of previously synthesized fluorides were mixed in specific molar ratios, pressed into tablets, and placed in quartz glass tubes, which were evacuated and then placed in reactors filled with helium and sealed. High-purity lanthanide oxides served as starting materials. Cerium (III) fluoride (CeF₃) was obtained from cerium dioxide (CeO₂) of the CeO-D grade (OST 48-195-81, Lviv Chemical Reagents Plant, Lviv, Ukraine) by treatment with ammonium fluoride and the addition of H₂O₂ as a reducing agent, followed by remelting in a graphite crucible. Europium (III) fluoride (EuF₃) was produced by treating europium (III) oxide of the EiO-Zh grade (OST 48-199-81, Lviv Chemical Reagents Plant, Lviv, Ukraine) with concentrated high-purity H₂F₂, followed by vacuum drying and high-temperature calcination in an inert gas atmosphere (He) (LLC “Ingas”, Mariupol, Ukraine).

The samples of the CeF₃–EuF₃ system were calcined in an inert atmosphere (He) at 1100 °C for 4 h in a vertical furnace, after which they were removed and cooled in air. After cooling, the samples of the system were ground and mixed with a NaCl–KCl salt solution of equimolar composition, with fluoride–salt mixture mass ratios of 1:9 and 2:8. The mixtures were again placed in quartz glass tubes of approximately 10 cm in height and 1 cm in diameter, which, in turn, were placed in a quartz glass reactor, evacuated, filled with inert gas (He), and then placed into a vertical furnace.

The heat treatment of samples in molten NaCl–KCl was carried out at a temperature of 750–800 °C (80–130 °C above the melting point of NaCl–KCl), at which the salt component remains in a molten state and becomes saturated with fluoride over 2–4 h. The furnace was then turned off, and after complete cooling, the tubes were removed. In the solidified salt melt, the dividing line between the bottom part (insoluble sediment) and the upper part (solidified solution-melt) was often clearly visible due to the significant difference in the density and refractive indices of the substances. In the case of the NaCl–KCl–CeF₃–EuF₃ (2:1) sample, an intermediate (middle) part of the solidified salt melt was also selected for study. The samples of solidified salt melts were colorless with faint, barely noticeable luminescence under direct sunlight.

### 2.2. X-Ray Diffraction Method

The X-ray diffraction (XRD) analysis of the products was conducted on a DRON-3M diffractometer with CuKα radiation (0.15418 nm) using the powder method. XRD images were captured with Bragg–Brentano focusing on the angular range of 10–80°, with a step size of 0.5° and an exposure time of 1 s. The dimensions of the Soller slits were 0.02/1.2/0.25 mm, and the device error was 0.01%.

To identify the phase composition of the synthesis products, the diffractogram was processed using the Match! Crystal Impact software version 3.3 with the FullProf toolbar [21] and the SCDD PDF-2 and COD (Crystallography Open Database) databases.

The Rietveld method and the Jana software version 2020 [22] were employed to quantify the phase content in the sample. Calculations were based on X-ray data from the Crystallography Open Database (COD) [23] for the following compounds: NaCl, KCl, CeF₃, EuF₂, and EuF₃. The calculation error did not exceed 2.5%.

### 2.3. Spectroscopic Methods

The following spectroscopic methods were employed: IR transmission spectroscopy, diffuse reflection spectroscopy (DR), and luminescence spectroscopy.

#### 2.3.1. IR Transmittance Spectroscopy

IR spectra were recorded in the coordinates T = f($\tilde{\nu }$), where T represents transmission and $\tilde{\nu }$ denotes wave number, using a Fourier-transform IR Frontier Perkin-Elmer spectrophotometer (Perkin-Elmer Inc., Waltham, MA, USA) in the wave number range of 4000–200 cm⁻^1^. CsI-based samples were prepared according to standard techniques. For IR transmission spectroscopy, the spectral range was 4000–200 cm⁻^1^, with an instrument resolution better than 0.4 cm⁻^1^; wavelength reproducibility was approximately 0.008 cm⁻^1^; and the signal–noise ratio exceeded 50,000:1.

#### 2.3.2. Diffuse Reflectance Spectroscopy

Diffuse reflectance spectra were measured using a Perkin Elmer Double Beam Scanning UV/VIS/NIR- Spectrophotometer Lambda 9 (PerkinElmer, Inc., Waltham, MA, USA) over the range of 200–2500 nm as a function of the following:

*F(R)* = *f*(*λ*) = (1−*R*)^2^/2*R* = *k*/s, (2)

which represents the Kubelka–Munk function, *R* is the relative reflectance, and *k* and *s* denote the absorption and scattering coefficients, respectively.

For diffuse reflectance spectroscopy, the accuracy of wavelength axis calibration is ±0.2 nm for the UV and visible ranges and ±0.8 nm for the near-IR range. Wavelength measurement reproducibility is ±0.05 nm for the UV and visible ranges and ±0.2 nm for the near-IR range. Measurement errors on the absorption scale (Kubelka–Munk function, F(R)) include the errors associated with scattered light, amounting to 0.0001 for the UV range and 0.002 at 1690 nm.

#### 2.3.3. Luminescence Spectroscopy

The spectra of luminescence and excitation were recorded on a Fluorolog FL3-22 (HORIBA Jobin Yvon Inc, Longjumeau Cedex, France) spectrofluorimeter. A 450 W Xenon lamp (model 1907) served as the excitation source. For the visible region of the spectrum, an R928P photomultiplier was used as the radiation detector. The excitation wavelength range was from 240 to 600 nm, and the luminescence wavelength range was from 290 to 850 nm. The photomultiplier registered radiation intensity by counting individual photons of light, maintaining proportionality within a range of 1000 to 2,000,000 counts per second (CPS). Based on this sensitivity range, the input and output slits of the device were adjusted accordingly. The luminescence measurements were conducted in a specialized cuvette for solid powder materials with a depth of 1.5 mm and a surface area of 70 mm^2^. Before recording the luminescence spectrum of the crystalline sample, the sample was ground into a uniform fine powder. The integrated luminescence intensity was determined from the area corresponding to the emission band using the ORIGIN8 software, and the emission wavelength was converted into wave numbers.

To measure the Eu^3^⁺ luminescence lifetime, the Xenon lamp was operated in pulse mode with a pulse duration of 3 μs. To measure the Eu^2^⁺ luminescence lifetime, an ultraviolet NanoLED with an excitation wavelength (λ_exc._) of 330 nm was used featuring a pulse width of 1.4 ns and a pulse frequency of 25 kHz.

## 3. Results and Discussion

### 3.1. X-Ray Diffractogram

**Figure 1:**
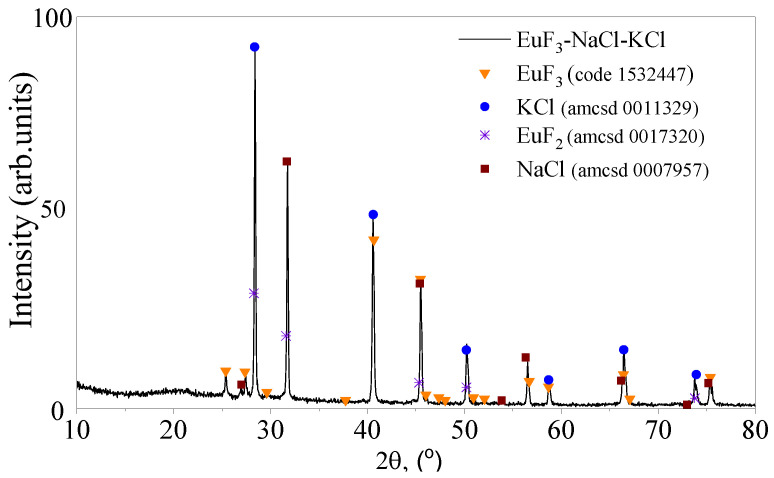
Diffractogram of solidified NaCl–KCl–EuF_3_ solution-melt.

The diffractogram obtained for the solidified melt solution NaCl–KCl–EuF_3_ (Figure 1) is quite similar to the diffractogram of NaCl–KCl–CeF_3_, which indicates the insignificant content of the dispersed phase (EuF_3_ and CeF_3_) and the significant predominance of the salt matrix.

The composition of EuF_3_ in the upper part of the solidified melt, according to XRD data, was 2.0 Quant(%), EuF_2_ 0.5 Quant(%), KCl 51 Quant(%), and NaCl 46.5 Quant(%).

### 3.2. Results of Spectroscopic Studies

#### 3.2.1. Results of IR Transmission Spectroscopy

**Figure 2:**
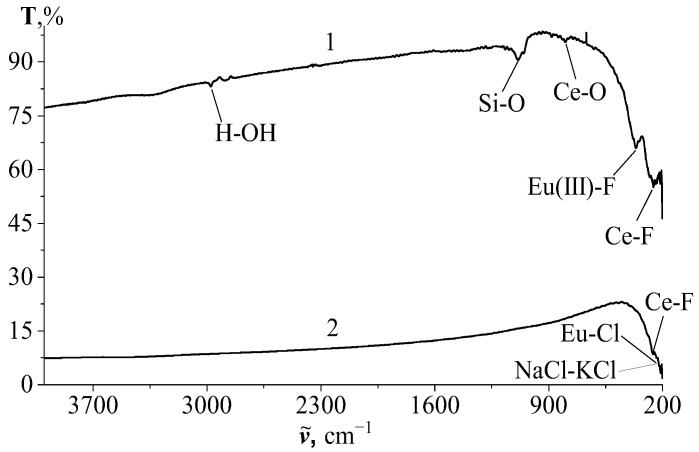
IR transmission spectra of system samples: 1—CeF_3_–EuF_3_ after calcination; 2—NaCl–KCl–CeF_3_–EuF_3_ (upper part of the solidified salt melt).

The IR transmission spectra (Figure 2, curve 1) of a CeF₃ + EuF₃ system sample in the range of 250–500 cm⁻^1^ display two peaks corresponding to the vibrations of Ce–F and Eu–F bonds. A band observed in the region of 1100–1200 cm⁻^1^ likely appeared due to Si–O impurities introduced during the calcination of the sample in a quartz glass vessel. A less pronounced band at approximately 800 cm⁻^1^ likely corresponds to the vibrations of Ce–O bonds, which arise as a result of the pyro-hydrolysis of one of the reaction products, specifically CeF₄.

The transmittance level of the sample is notably high, ranging from 75% to 95%, which indicates its high crystallinity. The level of transmittance of the sample is very high—75–95%, which indicates its high crystallinity.

In the samples containing a chloride melt as a base, i.e., a solidified solution-melt, the IR transmission spectrum shows the most prominent bands corresponding to the vibrations of the base bonds, NaCl and KCl, in the region of approximately 210–220 cm⁻^1^. Conversely, the bands corresponding to the soluble substances are much weaker (Figure 2, curve 2).

Due to the significantly lower heat treatment temperature of the salt melt samples, they lack the previously mentioned Si–O vibration band. The transparency of the samples in the NaCl–KCl–CeF₃–EuF₃ system is substantially lower, which likely indicates the presence of an ultra-microdisperse composition in the solidified melts.

#### 3.2.2. Results of Diffuse Reflectance Spectroscopy

**Figure 3:**
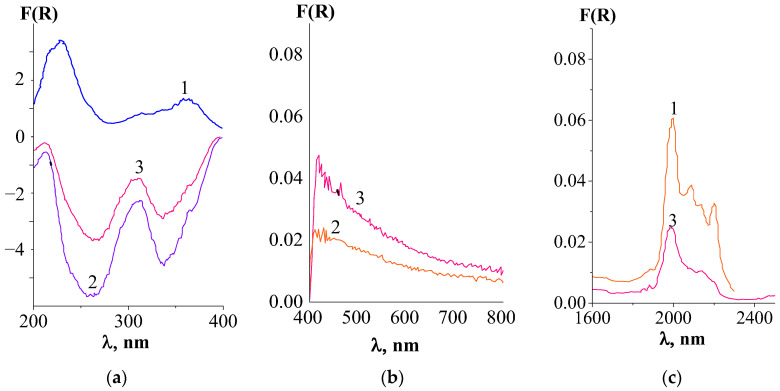
Diffuse reflectance spectra ((**a**) –UV, (**b**)–visible, and (**c**)–near-IR ranges) of system samples: 1–CeF_3_–EuF_3_ after calcination; 2–NaCl–KCl–CeF_3_–EuF_3_ (the upper part of the solidified salt melt); 3–NaCl–KCl–CeF_3_–EuF_3_ (insoluble sediment).

The analysis of the diffuse reflection spectra of the samples within the studied system generally confirms the predicted nature of the interaction. In particular, on the spectral dependence *F(R)* = *f*(λ) for the CeF₃–EuF₃ sample in the near-IR range (1900–2300 nm), weak remnants of the band corresponding to 4f-4f electronic transitions in Eu^3^⁺ ions were detected (Figure 3c, curve 1). The intensity of this residual band is 20–30 times lower compared to that in the original EuF₃ compound, indicating a significant decrease in the content or even the possible disappearance of the phase with this composition. Instead, an absorption feature appears in the UV range of the spectrum, consisting of two broad, high-intensity bands characteristic of 4f-5d electronic transitions in Eu^2^⁺ ions, partially overlapping with Ce^3^⁺ → Ce⁴⁺ charge transfer bands (Figure 3a, curve 1). This suggests that the EuCeF₆ compound, with a possible hexagonal local symmetry, may form within the system. More specifically, this could involve EuF₂, which transitions into the NaCl–KCl melt upon interaction.

The diffuse reflectance spectrum of the NaCl–KCl–CeF₃–EuF₃ solidified melt in the UV range (Figure 3a, curves 2 and 3) appears as a band with negative absorption, consisting of two peaks, which manifests as luminescence in the near-visible range of the spectrum. The intensity (depth) of the peaks in the homogeneous solidified salt melt (the upper part of the solidified melt) is more than twice that of the insoluble sediment. Notably, for the upper part of the solidified salt melt in the near-IR range, no peaks or absorption bands are observed. This finding supports the presence of only Eu(II) compounds and the absence of Eu(III) compounds.

The diffuse reflectance spectrum of the insoluble sediment in the near-IR range shows a broad, weakly resolved absorption band corresponding to 4f-4f electronic transitions in Eu^3^⁺ ions with very low intensity (almost 2.5 times weaker than that in the CeF₃–EuF₃ sample) (Figure 3c, curve 3). For Ce(III) and Ce(IV) compounds, it is challenging to confirm their presence or absence based on the diffuse reflectance spectra alone.

#### 3.2.3. Results of Luminescence Spectroscopy

Photoluminescence spectroscopy was employed to identify and detect Eu^3^⁺, Eu^2^⁺, and Ce^3^⁺ ions, as well as to provide a qualitative comparative assessment of their relative concentrations using the “more-less” principle based on the presence of characteristic emission bands resulting from 4f-4f (Eu^3^⁺) and 4f-5d (Eu^2^⁺ and Ce^3^⁺) electronic transitions [24,25,26].

By analyzing the changes in the spectra (such as the presence or shift in emission bands, their splitting, and intensity), it is possible to evaluate the processes occurring in the systems under study, specifically in the CeF₃–EuF₃ samples with varying component ratios synthesized in a melt solution of an equimolar NaCl–KCl mixture.

**Figure 4:**
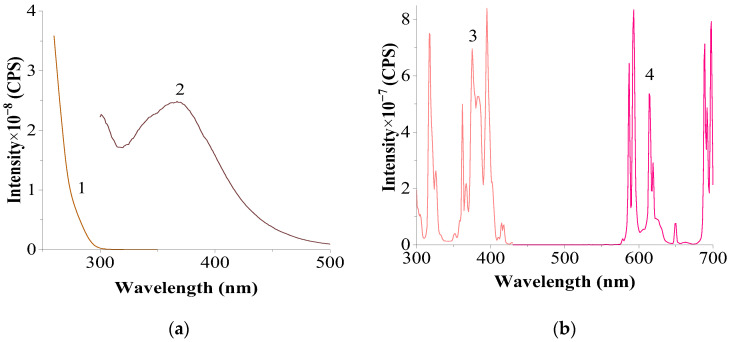
Excitation and luminescence spectra of the original CeF_3_ (**a**) and EuF_3_ (**b**) samples. (**a**) (slits 2.0–2.0 nm): 1—excitation spectrum of Ce^3+^, recorded at λ_em_. = 367 nm; 2—luminescence spectrum of Ce^3+^ at λ_exc_. = 265 nm; (**b**) (slits 1.5–1.5 nm): 3—excitation spectrum of Eu^3+^, recorded at λ_em_. = 593 nm; 4—luminescence spectrum of Eu^3+^, recorded at λ_exc._ = 395 nm.

The emission and luminescence spectra of the studied systems were compared with the spectra of several original reference samples. Figure 4 presents the excitation and luminescence spectra of the original CeF₃ and EuF₃.

Several characteristic bands with a maximum at 395 nm, the most intense among them, are observed in the excitation spectrum of EuF₃, which, with certain approximations, can be considered analogous to the absorption (diffuse reflectance) spectrum recorded at λ_em._ = 593 nm. A similar maximum is observed in the diffuse reflectance spectrum of EuF₃ (λ_max._ = 393.7 nm) [27]. In the luminescence spectrum, peaks characteristic of Eu^3^⁺, corresponding to the 5D₀ → 7F₁ (λ_max._ 587, 593 nm), 5D₀ → 7F₂ (λ_max._ 615, 620 nm), 5D₀ → 7F₃ (λ_max._ 649 nm), and 5D₀ → 7F₄ transitions (λ_max._ 689, 692, 698 nm), are observed, showing characteristic splitting and relative peak intensities.

The luminescence spectrum of CeF₃ shows a broad, diffuse band with a blurred maximum at 365–372 nm, whose position, according to previous studies, does not depend on the excitation wavelength.

As expected, the luminescence spectrum of the mechanical mixture CeF₃:EuF_3_ (1:1) (not shown) [17] includes the emission bands of Ce^3^⁺ and Eu^3^⁺. However, a minimum at around 398 nm is observed on the spectral curve, attributed to 4f-5d electronic transitions in the Ce^3^⁺ ion, with the primary maximum of the band hypsochromically shifted (λ_max._ = 336 nm) compared to the spectrum of pure CeF₃. A similar pattern is observed in the spectrum (not shown) [17] of a CeF₃:EuF_3_ mechanical mixture with a 1:2 component ratio, where a minimum in the Ce^3^⁺ emission curve (λ_min._ = 398 nm) and a hypsochromic shift in the primary maximum (λ_max._ = 332 nm) are also detected. The Eu^2^⁺ peak is not observed. The positions of characteristic peaks in the excitation spectrum of the Eu^3^⁺ sample (317, 374, and 394 nm) correlate well with the positions of the bands in its diffuse reflectance spectrum (318, 375, and 385 nm).

**Figure 5:**
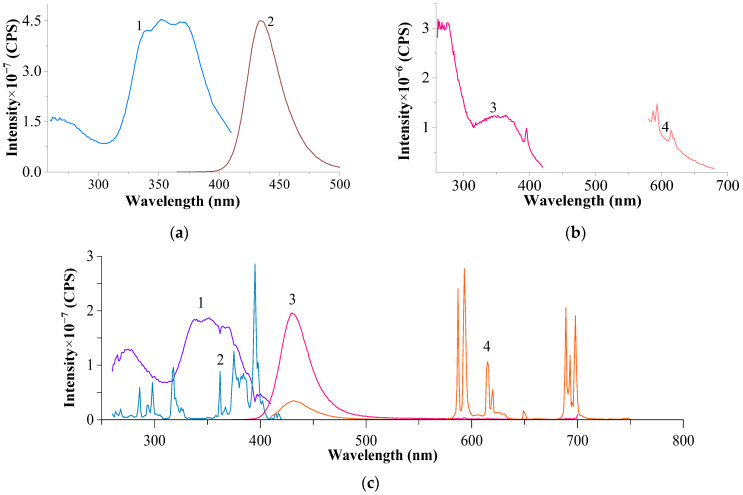
Excitation and luminescence spectra of the upper (**a**,**b**) and lower (**c**) parts of the EuF_3_ melt in NaCl–KCl (1:9): a (slits 0.6–0.6 nm): 1—excitation spectrum of Eu^2+^ at λ_em._ = 435 nm; 2—luminescence spectrum of Eu^2+^ at λ_exc._ = 352 nm; b (slits 2.0–2.0 nm): 3—excitation spectrum of Eu^3+^ at λ_em._ = 615 nm; 4—luminescence spectrum of Eu^3+^ at λ_exc._ = 394 nm; c (slits 0.6–0.6 nm): 1—excitation spectrum of Eu^2+^ at λ_em._ = 435 nm; 2—excitation spectrum of Eu^3+^ at λ_em._ = 593 nm; 3—luminescence spectrum of Eu^2+^ at λ_exc._ = 352 nm; 4—luminescence spectrum of Eu^3+^ at λ_exc._ = 395 nm.

The spectra of NaCl–KCl–CeF₃ (not shown) [19] and NaCl–KCl–EuF₃ systems with a fluoride–melt ratio of 1:9 were recorded (Figure 5). In the excitation spectrum of the EuF₃ sample (λ_em._ = 435 nm), a broad, intense band with three maxima is recorded, which, as shown below, is characteristic of all the samples containing Eu^2^⁺. The most intense peak for the analyzed system is observed at λ_exc._ = 352 nm. Notably, this band coincides with a minimum in the region of negative F(R) values on the diffuse reflectance spectrum [28]. The luminescence spectra of the upper and bottom parts of the sample indicate the formation of Eu^2^⁺ ions in the NaCl–KCl–EuF₃ system, evidenced by the presence of an intense band with a maximum at 434 nm.

The presence of a significantly smaller amount of Eu^3+^ ions in the upper part compared to the bottom part (insoluble sediment) confirms the proposed mechanism of the interaction of EuF_3_ with the melt. It consists in the fact that when EuF_3_ is dissolved in the NaCl–KCl melt, a redox reaction occurs according to the following scheme:

(3) $${EuF}_{3}+3NaCl\overset{T,He}{\to }{EuCl}_{2}+\frac{1}{2}{Cl}_{2}↑+3NaF,$$

and subsequently [29]:

(4) $$2KCl+{EuCl}_{2}\overset{T,He}{\to }K_{2}[{EuCl}_{4}].$$

The luminescence spectrum of the upper part of the NaCl–KCl–CeF₃ sample is similar to that of the original CeF₃, showing the Ce^3^⁺ ion band (λ_max._ = 368 nm) with greater intensity than the band of the original fluoride (2.03 × 10⁸ and 1.4 × 10⁸ CPS, respectively), which may indicate reduction upon dissolution and, correspondingly, an effect of concentration quenching. It was also of interest to determine how the addition of Ce^3^⁺ ions to the salt melt influences the emission intensity of Eu^2^⁺ ions.

**Figure 6:**
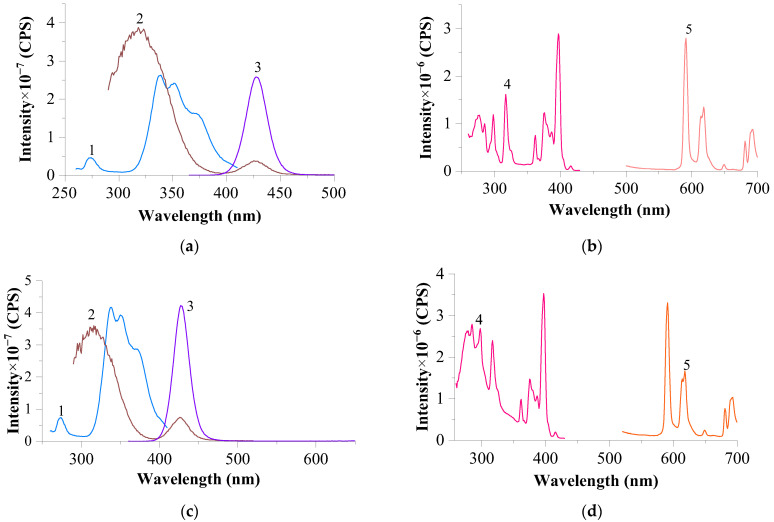
Excitation and luminescence spectra of the upper part of the melt (**a**,**b**) and the bottom part (**c**,**d**) of the melt of a mechanical mixture of CeF_3_–EuF_3_ (1:1) in NaCl–KCl (1:9): a (slits 0.6–0.6 nm): 1—excitation spectrum of Eu^2+^ at λ_em._ = 428 nm; 2—luminescence spectrum of Ce^3+^ at λ_exc._ = 274 nm; 3—luminescence spectrum of Eu^2+^ at λ_exc._ = 338 nm; b (slits 3.0–3.0 nm): 4—excitation spectrum of Eu^3+^ at λ_em._ = 591 nm; 5—luminescence spectrum of Eu^3+^ at λ_exc._ = 396 nm; c (slits 0.6–0.6 nm): 1—excitation spectrum of Eu^2+^ at λ_em._ = 428 nm; 2—luminescence spectrum of Ce^3+^ at λ_exc._ = 273 nm; 3—luminescence spectrum of Eu^2+^ λ_exc._ = 340 nm; d (slits 3.0–3.0 nm): 4—excitation spectrum of Eu^3+^ at λ_em._ = 591 nm; 5—luminescence spectra of Eu^3+^ at λ_exc._ = 397 nm.

To determine the optimal synthesis conditions and composition, various parameters were adjusted. Figure 6 presents the excitation and luminescence spectra of the sample obtained by dissolving a CeF₃:EuF_3_ (1:1) mechanical mixture in NaCl–KCl at 800 °C with a holding time of 4 h.

The maximum in the broad diffuse band (Eu^2^⁺) is fixed at 340 nm in the excitation spectra recorded at λ_em._ = 428 nm. Additionally, a maximum at 274 nm is observed, corresponding to the excitation spectrum of Ce^3^⁺. The following emission bands appear in the luminescence spectra: an intense Ce^3^⁺ band with a hypsochromic shift in the emission maximum at λ_max._ = 318 nm, an intense Eu^2^⁺ band with λ_max._ = 427 nm, and low-intensity Eu^3^⁺ bands corresponding to the 5D₀ → 7F₁ and 5D₀ → 7F₄ electronic transitions, exhibiting characteristic splitting.

The spectra of the bottom and upper parts of the sample are identical in terms of the nature and position of the maxima, differing only slightly in band intensity. This suggests that the sample of the solidified salt melt system is relatively homogeneous.

**Figure 7:**
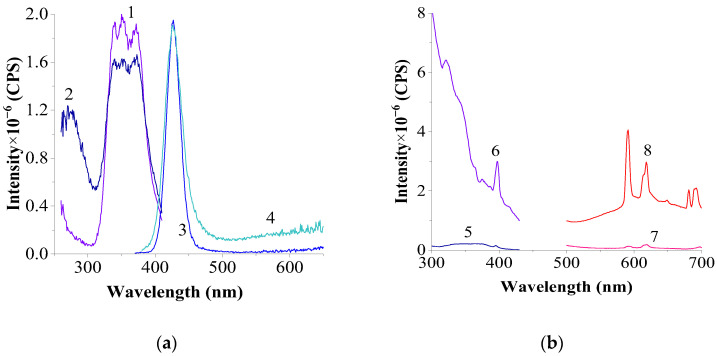
Excitation and luminescence spectra of the bottom and upper parts of the sample CeF_3_–EuF_3_ (1:1) in NaCl–KCl (1:9): (**a**)—excitation and luminescence spectra of Eu^2+^, slits 0.6–0.6 nm; 1—excitation spectrum at λ_em._ = 428 nm (upper part), 2—λ_em._ = 426 nm (bottom part); 3—luminescence spectrum at λ_exc._ = 352 nm (upper part), 4—λ_exc._ = 352 nm (bottom part); (**b**)—excitation and luminescence spectra of Eu^3+^, slits 3.0–3.0 nm; 5—excitation spectrum at λ_em_. = 613 nm (upper part), 6—λ_em._ = 618 nm (bottom part); 7—luminescence spectrum at λ_exc._ = 395 nm (upper part), 8—λ_exc._ = 397 nm (bottom part).

Figure 7 shows the spectra obtained under similar conditions, but from a CeF₃ and EuF₃ mixture calcined at 1100 °C. The excitation spectra of Eu^2^⁺ show three characteristic maxima, with the most intense recorded at 350 nm (upper part of the sample) and 373 nm (bottom part). In the bottom part spectrum, an excitation band of Ce^3^⁺ (λ_max._ = 270 nm) is observed, which is nearly absent in the spectrum of the upper part. The excitation spectrum of Eu^3+^ resembles a similar spectrum of the calcined CeF_3_–EuF_3_ mixture.

No Ce^3^⁺ bands were observed in the luminescence spectra of either the bottom or upper parts; however, the bands characteristic of Eu^2^⁺ and Eu^3^⁺ are present, with the intensity of Eu^3^⁺ emission bands being significantly higher in the bottom part. This suggests that under the selected synthesis conditions, the redox reactions are shifted toward the formation of Eu^2^⁺. If we consider the possibility of a reaction occurring during calcination according to the following scheme:

(5) $${CeF}_{3}+{EuF}_{3}\overset{T,He}{\to }{CeF}_{4}↑+{EuF}_{2},$$

and then,

(6) $${EuF}_{2}+{CeF}_{4}\overset{T,He}{\to }{EuCeF}_{6},$$

it becomes clear where CeF_3_ could go, and therefore Ce^3+^ ions from the melt solution.

To further investigate the composition, the original CeF₃–EuF₃ mixture ratio was slightly adjusted from 1:1 to 1:2.

**Figure 8:**
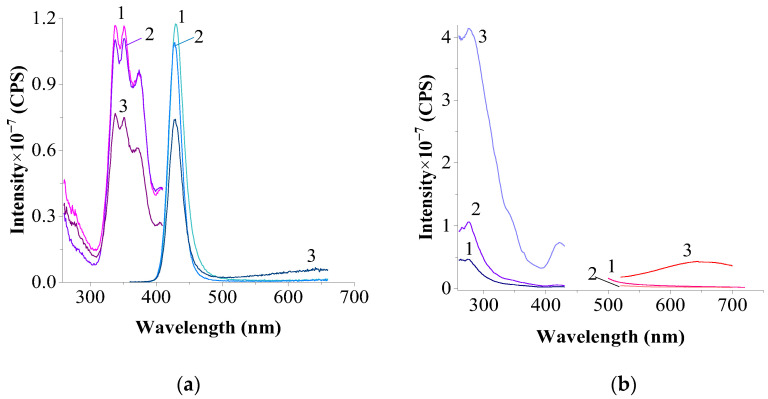
Excitation and luminescence spectra of the upper (1), middle (2), and bottom (3) parts of the sample CeF_3_–EuF_3_ (1:2) in NaCl–KCl (1:9): (**a**)—excitation and luminescence spectra of Eu^2+^: λ_em._ = 429 nm; λ_exc._ = 338 nm; slits 0.6–0.6 nm; (**b**)—excitation and luminescence spectra of Eu^3+^: λ_em._ = 591 nm; λ_exc._ = 397 nm; slits 3.0–3.0 nm.

Figure 8 shows the luminescence spectra of the CeF₃:EuF_3_ (1:2) system (calcined at 1100 °C). The excitation and luminescence spectra of the upper and middle parts of the sample nearly coincide, while the spectra of the bottom part exhibit a similar profile, except for the luminescence spectrum at λ_exc._ = 398 nm in the bottom part. Notably, the bands associated with Eu^3^⁺ ions are absent in both the excitation and luminescence spectra. Clearly defined bands of Eu^2^⁺ and the Ce^3^⁺ ion (as indicated by the excitation spectra) appear to be present only in trace amounts.

**Figure 9:**
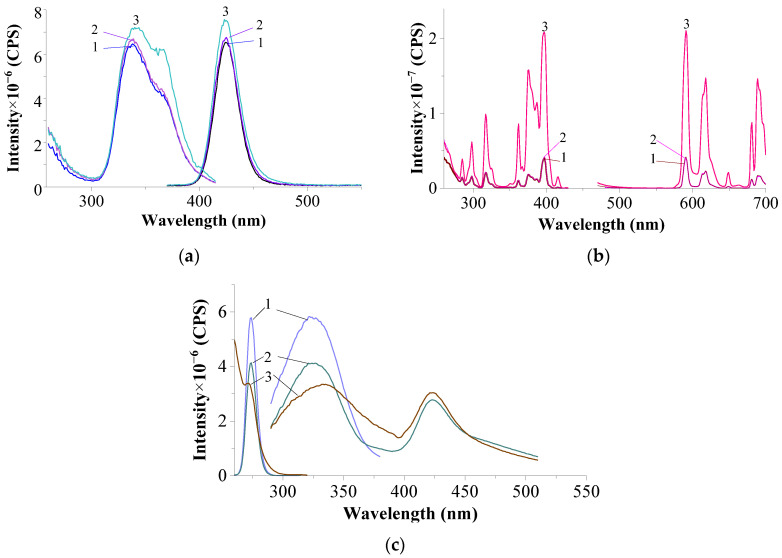
Excitation and luminescence spectra of the upper (1), middle (2), and bottom (3) parts of the sample CeF_3_–EuF_3_ (1:2) in NaCl–KCl (2:8), 750 °C for 2 h in a vacuum: (**a**)—excitation and luminescence spectra of Eu^2+^: λ_em._ = 425 nm; λ_exc._ = 338 nm; slits 0.6–0.6 nm; (**b**)—excitation and luminescence spectra of Eu^3+^: λ_em._ = 591 nm; λ_exc._ = 397 nm; slits 3.0–3.0 nm; (**c**)—excitation and luminescence spectra of Ce^3+^: λ_em._ = 325 nm; λ_exc._ = 273 nm; slits 3.0–3.0 nm.

According to Figure 9, for a CeF₃–EuF₃ (1:2) sample prepared from a mechanical mixture under vacuum (2 h) at 750 °C with a charge ratio of 2:8 (fluoride mixture to solidified salt melt), the typical excitation spectra of Ce^3^⁺ (except in the bottom part) and Eu^3^⁺ are observed.

A single maximum (two in the bottom part) appears at 338–340 nm within the broad diffuse band of the Eu^2^⁺ excitation spectrum. In the emission spectra recorded at λ_exc._ = 273 nm (optimal for Ce^3^⁺), both cerium and Eu^2^⁺ bands are detected, with Eu^3^⁺ emission band intensity highest in the bottom part.

For a CeF₃:EuF_3_ (2:1) sample prepared from a mechanical mixture under vacuum (2 h) at 750 °C with a fluoride–salt melt ratio of 2:8 (Figure 9), a characteristic Ce^3^⁺ band is observed only in the excitation spectrum of the upper part of the solidified salt melt and is absent in the bottom part.

In the Eu^3^⁺ excitation range, the intensity of characteristic bands is considerably higher in the bottom part. In the emission spectra recorded at λ_exc._ = 273 nm, the characteristic Ce^3^⁺ band is weak and appears only in the upper part, although Eu^2^⁺ bands are present in both parts. The intensity of these Eu^2^⁺ bands is higher (or, in the case of the bottom part, nearly equal) to that of similar bands recorded at the excitation wavelengths characteristic of divalent europium. The intensity of Eu^3^⁺ emission bands is very low.

The position of the radiation maximum (${\bar{\lambda }}_{max.}$ = 430 nm) practically does not depend on the excitation wavelength in the investigated wavelength range.

The obtained data indicate that interaction within the system is most complete (with minimal luminescence intensity from Ce^3^⁺ and Eu^3^⁺ ions and maximal luminescence intensity from Eu^2^⁺ ions) when synthesis is conducted with a calcined mechanical mixture at 1100 °C and a salt melt ratio of 1:9. A similar degree of interaction occurs with a fluoride mechanical mixture to salt melt ratio of 2:8 at 750 °C under vacuum conditions. In both cases, the CeF₃:EuF_3_ ratio was maintained at 1:2. Given that the studied materials show promise as ultraviolet radiation detectors, Eu^2^⁺ luminescence spectra (from the upper part of the solidified salt melt) were recorded at different excitation wavelengths within the UV range. The results are presented in Table 1.

As shown in Table 1, when the sample is excited by radiation across almost the entire UVA-UVB wavelength range, intense Eu^2^⁺ ion luminescence is observed, reaching its peak values at λ_exc._ = 340–350 nm. At this point, both the maximum intensity (I_max._) and integrated intensity (I_int._) achieve their highest values in the excitation wavelength range of 330–375 nm. A qualitative correlation is observed between the luminescence excitation spectrum and the solar ultraviolet radiation spectrum [1].

The position of the emission maximum (${\bar{\lambda }}_{max.}$ = 430 nm) remains nearly constant across the investigated excitation wavelength range.

The luminescence lifetimes (τ) of Eu^2+^ and Eu^3+^, calculated from the decay curves for various samples, are presented in Table 2.

The luminescence lifetime of Eu^2^⁺ ions, as determined in this study, is approximately 1 µs, while for Eu^3^⁺ ions, it ranges from 700 to 1300 µs—over a thousand times longer. This observation aligns with the established theories regarding the mechanisms of excitation and emission for europium in different valence states.

## 4. Conclusions

The results from the diffuse reflectance spectroscopy and luminescence spectroscopy of the NaCl–KCl–CeF₃–EuF₃ system, particularly regarding the reduction of Eu(III) to Eu(II), show satisfactory agreement. The data indicate that the redox interaction between CeF₃ and EuF₃ in the NaCl–KCl melt is most complete with a fluoride–melt mass ratio of 1:9 following the preliminary calcination of the fluoride components at 1100 °C, or with a fluoride–melt ratio of 2:8 for the mechanical mixture of fluorides in the salt melt and a CeF₃ to EuF₃ ratio of 1:2. Under these conditions, the maximum luminescence intensity of Eu^2^⁺ ions is achieved at an excitation wavelength of 340–350 nm, while luminescence from Eu^3^⁺ and Ce^3^⁺ ions is minimized.

This study highlights the potential of these materials for ultraviolet solar radiation detectors. A key advantage of the materials in this system is their lack of hygroscopicity, which contrasts with the highly hygroscopic matrices currently used, such as CaCl₂ and SrCl₂, and EuCl₂ as an activator. The absence of hygroscopicity in this system (with NaCl, KCl, EuF₃, and CeF₃ as the primary components) simplifies sample preparation, handling, and storage. One limitation of these materials, however, is the difficulty of obtaining a single-crystal sample due to system heterogeneity. Under prolonged operating conditions, the optimization of the system’s composition and structure may be necessary to achieve optimal detector performance. This limitation may be mitigated by employing a broad range of solid solutions within the NaCl–KCl system at high temperatures.

## 5. Patents

The method for obtaining the material for the detectors of solar ultraviolet radiation is protected by Ukrainian patent no. 152026.

## Figures and Tables

**Table 1:** Parameters of the luminescence spectra of Eu^2+^ in the upper part of the NaCl–KCl–CeF_3_–EuF_3_ system (1:2, fired at 1100 °C) with a fluoride–salt melt ratio of 1:9, slits 0.6–0.6 nm.

No pos.	Wavelength, nm		Intensity	
	λ_exc._	λ_max._	I_max._ × 10^–6^, CPS	I_int._ × 10^–7^, rel. u.
1	261	431	4.53	14.30
2	300	432	1.66	6.19
3	310	432	1.55	5.44
4	317	430	2.20	7.85
5	330	429	7.31	22.79
6	340	430	11.7	34.91
7	350	430	11.1	32.74
8	362	430	8.95	26.98
9	375	430	9.04	26.42
10	387	430	5.57	16.20
11	397	430	3.87	10.48

**Table 2:** Luminescence lifetime of Eu^2+^ and Eu^3+^ ions in the upper part of the samples synthesized from a mechanical mixture of fluorides by heat treatment in a NaCl–KCl melt, vacuum, 750 °C (λ_exc._ of Eu^2+^ = 338 nm; λ_exc_. of Eu^3+^ = 398 nm).

No pos.	Sample	Eu^2+^		Eu^3+^	
		λ_max._, nm	τ, µs	λ_max._, nm	τ, µs
1	CeF_3_–EuF_3_ (1:1) + NaCl–KCl (1:9)	428 *	1.07	591	1301
2	CeF_3_–EuF_3_ (1:2) + NaCl–KCl (2:8)	424 *	1.00	591	699 **
3	CeF_3_–EuF_3_ (2:1) + NaCl–KCl (2:8)	428 *	1.02	591	975 **

*—diode lamp; **—average of 2 measurements.

## Data Availability

The raw/processed data required to reproduce these findings can be obtained from the corresponding author (volchakganna@gmail.com) upon reasonable request.
